# Impact of positive end-expiratory pressure and recruitment maneuver on healthy lungs in dogs assessed by functional and anatomical monitoring methods

**DOI:** 10.3389/fvets.2025.1545683

**Published:** 2025-03-06

**Authors:** Martina Mosing, Andreas D. Waldmann, Thom C. Gent, Giselle Hosgood, Nadja S. Sieber-Ruckstuhl, Matthias Dennler, Peter Herrmann, Karin Unger

**Affiliations:** ^1^Clinical Department for Small Animals and Horses, Clinical Center for Small Animals, University of Veterinary Medicine, Vienna, Austria; ^2^Section of Anaesthesiology, Vetsuisse Faculty, University of Zurich, Zurich, Switzerland; ^3^Department of Anaesthesiology and Intensive Care Medicine, Rostock University Medical Centre, Rostock, Germany; ^4^College of Veterinary Medicine, School of Veterinary and Life Sciences, Murdoch University, Perth, WA, Australia; ^5^Clinic for Small Animal Internal Medicine, Vetsuisse Faculty, University of Zurich, Zurich, Switzerland; ^6^Clinic for Diagnostic Imaging, Vetsuisse Faculty, University of Zurich, Zurich, Switzerland; ^7^Department of Anaesthesiology, University Medical Center, Georg-August-University, Goettingen, Germany

**Keywords:** anesthesia, center of ventilation, dogs, short-term mechanical ventilation, overdistension

## Abstract

**Introduction:**

Atelectasis is a common occurrence during anesthesia, and positive end-expiratory pressure (PEEP) ventilation and recruitment maneuvers (RM) can be used to mitigate this. However, both techniques may be associated with side effects in healthy lungs, and close monitoring is indicated. This study aimed to evaluate the effects of PEEP and RM in healthy dogs and to compare functional lung monitoring methods by electrical impedance tomography (EIT), volumetric capnography (VCap), and blood gas analysis with the gold-standard anatomical monitoring provided by computed tomography (CT).

**Methods and materials:**

Nine healthy Beagle dogs underwent anesthesia and mechanical ventilation three times. After 35 min using zero end-expiratory pressure (ZEEP), CT images, VCap, EIT measurements, and arterial blood gas samples were taken. Thereafter, either (1) ZEEP was continued, (2) PEEP initiated or (3) an RM was performed followed by PEEP. Ten minutes after changing the ventilation mode all measurements were repeated. Only one ventilation mode was employed during each anesthesia.

**Results:**

During RM, we found a significant increase in the percentage of overaerated lung (V_hyper_) (*p* < 0.001), while the amount of normally aerated lung (V_normal_), poorly aerated lung and non-aerated lung decreased (*p* ≤ 0.001). VCap showed an increase in airway dead space (VD_aw_/VT) (*p* = 0.002), and a decrease in alveolar dead space (VD_alv_/VT_alv_). For PEEP, an increase in airway dead space (*p* = 0.003) was found. For both groups, the amount of carbon dioxide exhaled per breath (VTCO_2,br_) decreased (*p* = 0.001), and EIT showed a shift of the center of ventilation to the dependent lung areas (*p* = 0.021 and *p* = 0.046, respectively). Oxygenation was superior in RM compared to ZEEP (*p* = 0.033). The arterial partial pressure of carbon dioxide decreased in RM (*p* = 0.012). Positive associations were found between V_hyper_ and VD_aw_/VT (*p* = 0.004), V_hyper_ and VD_aw_/VT (*p* = 0.004), V_hyper_ and V_normal_ with VTCO_2,br_ (*p* = 0.002 for both). Negative associations were found between V_hyper_ and VD_alv_/VT_alv_ (*p* = 0.004) and non-dependent silent spaces (*p* = 0.050), and V_normal_ with oxygenation (*p* = 0.030).

**Conclusion:**

While RM may be effective in improving gas exchange, it appears to be not benign in healthy lungs, and PEEP might be the preferable strategy to avoid lung collapse during anesthesia. Functional monitoring – EIT, VCap, blood gas analysis – does not detect changes corresponding to anatomical findings on CT.

## 1 Introduction

Atelectasis is a common occurrence in anesthesia and has also been demonstrated in dogs with healthy lungs during mechanical ventilation (1). In humans, it is associated with increased post-operative morbidity (2). strategies may be employed to either correct or prevent atelectasis. Positive end expiratory pressure (PEEP) is intended to prevent the collapse of alveoli and therefore maintain gas exchange (3), while recruitment maneuvers aim to re-open collapsed alveoli (4). However, both techniques may be associated with overdistension of lung tissue resulting in inflammation in healthy lungs (5).

Computed tomography (CT) is the gold standard in anatomical monitoring of the lung status by assessing the amount of overinflated lung tissue and atelectasis (6) and is used to guide ventilation strategies in human medicine (7). However, CT does not allow breath-by-breath monitoring, is associated with radiation exposure and is not available at the bedside (6). Alternative monitoring methods are called for.

Volumetric capnography (VCap), during which the expiratory partial pressure of carbon dioxide (CO_2_) is plotted against the expired tidal volume, is a functional monitoring method to determine dead space on a breath-by-breath basis (8). It can be used to separate physiologic dead space into airway and alveolar dead space, the latter of which represents ventilated but non-perfused alveoli and can thus be considered a surrogate of functional overdistension of lung tissue (9).

Electrical impedance tomography (EIT) is a non-invasive technology for functional ventilation monitoring that uses electrical currents to create real-time images of tissues (10). Thoracic EIT has been used to investigate and monitor regional distribution of ventilation in conscious and anesthetized animals (11–18). Several parameters can be determined to describe ventilation distribution: the center of ventilation (CoV) is a representation of where in the thorax ventilation is focused. Silent spaces correspond to areas of poor ventilation and are considered to represent atelectasis if located in the dependent lung areas (dependent silent spaces, DSS), and hyperinflated lung tissue if located in the non-dependent areas (non-dependent silent spaces, NSS) (10).

Blood gas analysis is the gold standard to functionally assess global gas exchange in critically ill and anesthetized patients. Arterial partial pressure of oxygen (PaO_2_) and carbon dioxide (PaCO_2_) are used as the main outcome values to determine respiratory status or the efficiency of a specific ventilation mode (19).

To monitor for complications associated with PEEP and RM, both VCap and/or EIT have been used in humans and dogs (4, 12, 13, 20–22). However, the information obtained by these two functional monitoring methods has not been compared to the anatomical situation revealed by CT in healthy anesthetized subjects. This information is crucial to evaluate the interpretation of EIT and VCap variables as an alternative monitoring tool to CT during mechanical ventilation. This study had two aims: Firstly, to determine the effect of PEEP and RM on healthy lungs in dogs and secondly to compare the outcome of monitoring functional lung status by EIT, VCap and blood gas analysis with the anatomical status assessed by CT.

## 2 Materials and methods

### 2.1 Animals

Nine healthy sexually intact Beagle dogs weighing between 9.1 and 14.7 kg (mean 13 ± 1.78 kg) and aged between 28 and 33 months (median 33 months, interquartile range 32–33 months) were used in this study. All dogs were considered healthy based on physical examination, routine hematology and biochemistry, and urinalysis. The dogs were housed at a university facility in standard kennels in groups of 2 to 4, fed dry adult maintenance dog food, and had free access to water. Animals were fasted overnight, and water was withheld 2 h before anesthesia induction. The study was approved by the Swiss Federal Ethics Committee for animal research of the Canton of Zurich (Nr. 185/2012). Each dog was anesthetized three times and only one ventilation strategy was used during each anesthesia event.

### 2.2 Anesthesia

Animals were premedicated with acepromazine (0.03 mg kg^−1^ IM, Prequillan, Fatro) and methadone (0.2 mg kg^−1^ IM, Methadon, Streuli Pharma). After placement of an indwelling intravenous catheter, carprofen was administered (4 mg kg^−1^ IV, Norocarp, Norbrook) and anesthesia was induced with propofol (Propofol 1% MCT, Fresenius Kabi). The trachea was intubated with a cuffed endotracheal tube (Surgivet, Smiths Medical, United States), which was then connected to a rebreathing circuit. Animals were placed in dorsal recumbency, and anesthesia was maintained with sevoflurane (E_T_ 2.0%, Sevorane, Abbott) and rocuronium was administered (0.3 mg kg^−1^) IV after induction and immediately before initiation of the study intervention [T0] (Esmeron, MSD AG). Lungs were ventilated (Datex S/5 Avance) using a volume-controlled mode with a tidal volume (VT) of 10 mL kg^−1^, respiratory rate of 25 bpm and an inspiratory-to-expiratory (I:E) ratio of 1:2, and initially ZEEP in all groups. Fraction of inspired oxygen (FiO_2_) was set at 0.21 for the first 5 min as part of a parallel study (23) and changed to 1.0 thereafter. Respiratory rate was adjusted to maintain end-tidal CO_2_ between 4.7 and 5.3 kPa (35–40 mmHg). An arterial catheter was placed in the dorsal metatarsal artery for blood pressure monitoring and blood sampling. Lactated Ringer’s solution was administered intravenously at a rate of 10 mL kg^−1^ h^−1^ throughout anesthesia. After each anesthesia event and data collection related to the study presented herein, anesthesia was continued for a subsequent imaging study (23). Thereafter, the dogs were allowed to recover.

### 2.3 Ventilation strategies and experimental design

**Figure 1:**
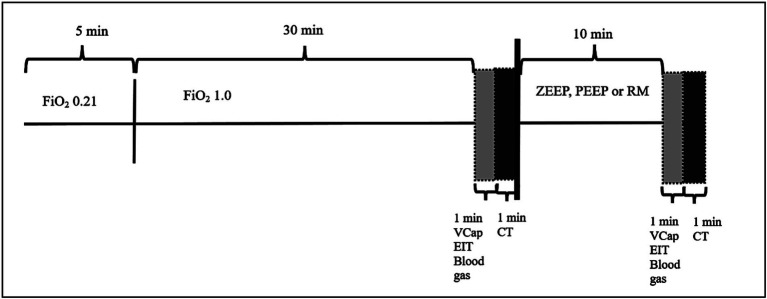
Experimental timeline.

The experiment was designed as a randomized cross-over study. Each dog was anesthetized three times with at least one-week washout period between each event. Only one ventilation technique was used during each anesthesia event in a randomized order. After 5 min of ventilation at an FiO_2_ of 0.21, FiO_2_ was increased to 1.0 and dogs were ventilated with ZEEP for 32 min (total time of ZEEP ventilation for all groups: 37 min, see Figure 1). Thereafter, ventilatory setting were as follows:

1. ZEEP: Volume-controlled ventilation with ZEEP for 12 min (control group).
2. PEEP: Volume-controlled ventilation with a PEEP of 5 cmH_2_O for 12 min (PEEP group).
3. RM: Recruitment maneuver followed by volume-controlled ventilation with a PEEP of 5 cmH_2_O for 12 min (RM group). The recruitment maneuver was performed in a manner similar to what has previously been described (24). Briefly, dogs were switched to pressure-controlled ventilation with a fixed respiratory rate of 25 breaths per minute. Peak inspiratory pressure and PEEP were increased and then decreased in a step-wise approach after the following schematic: 5 breaths at 10/0; 5 breaths at 15/5; 5 breaths at 20/10; 1 breath at 25/15; 1 breath at 30/15; 10 breaths at 40/15; 1 breath at 30/15, 1 breath at 25/15, 5 breaths at 20/10, and 5 breaths at 15/5. Thereafter, ventilation was switched back to volume-controlled and a PEEP of 5 cmH_2_O maintained until the end of the study period.

### 2.4 Measurements

After 35 of the 37 min of ZEEP ventilation had elapsed, VCap and EIT data were collected over 1 min, blood gas samples were taken, and a CT was performed over another minute (total duration of ZEEP ventilation: 37 min), before one of the three ventilation modes (ZEEP, PEP, RM) was initiated as described above. After 47 min of anesthesia (10 min after T0 measurements were completed) all measurements were repeated (Figure 1) (T1).

**Figure 2:**
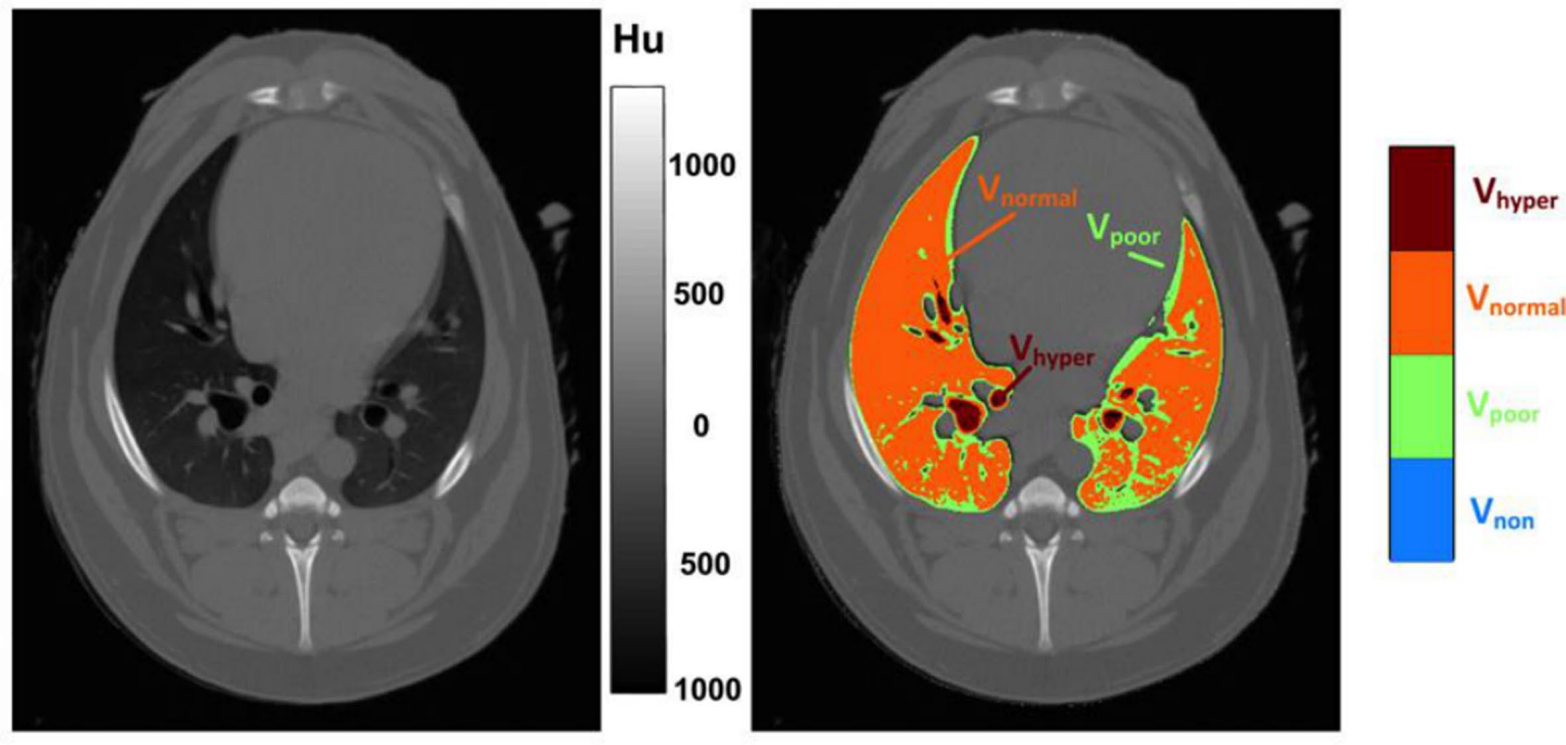
Graphical illustration of the analysis of chest computed tomography (CT). After CT images were obtained, the regions of interest (lung parenchyma and airways) were manually delineated using a software (Maluna version 3.14, University of Göttingen), and analyzed and color coded for degree of aeration. Left panel: Original CT image. Right panel; CT image after editing by software. Hyper- (V_hyper_), normally- (V_normal_), poorly- (V_poor_) and non-(V_non_) aerated lung volume. HU: Hounsfield Units.

**Figure 3:**
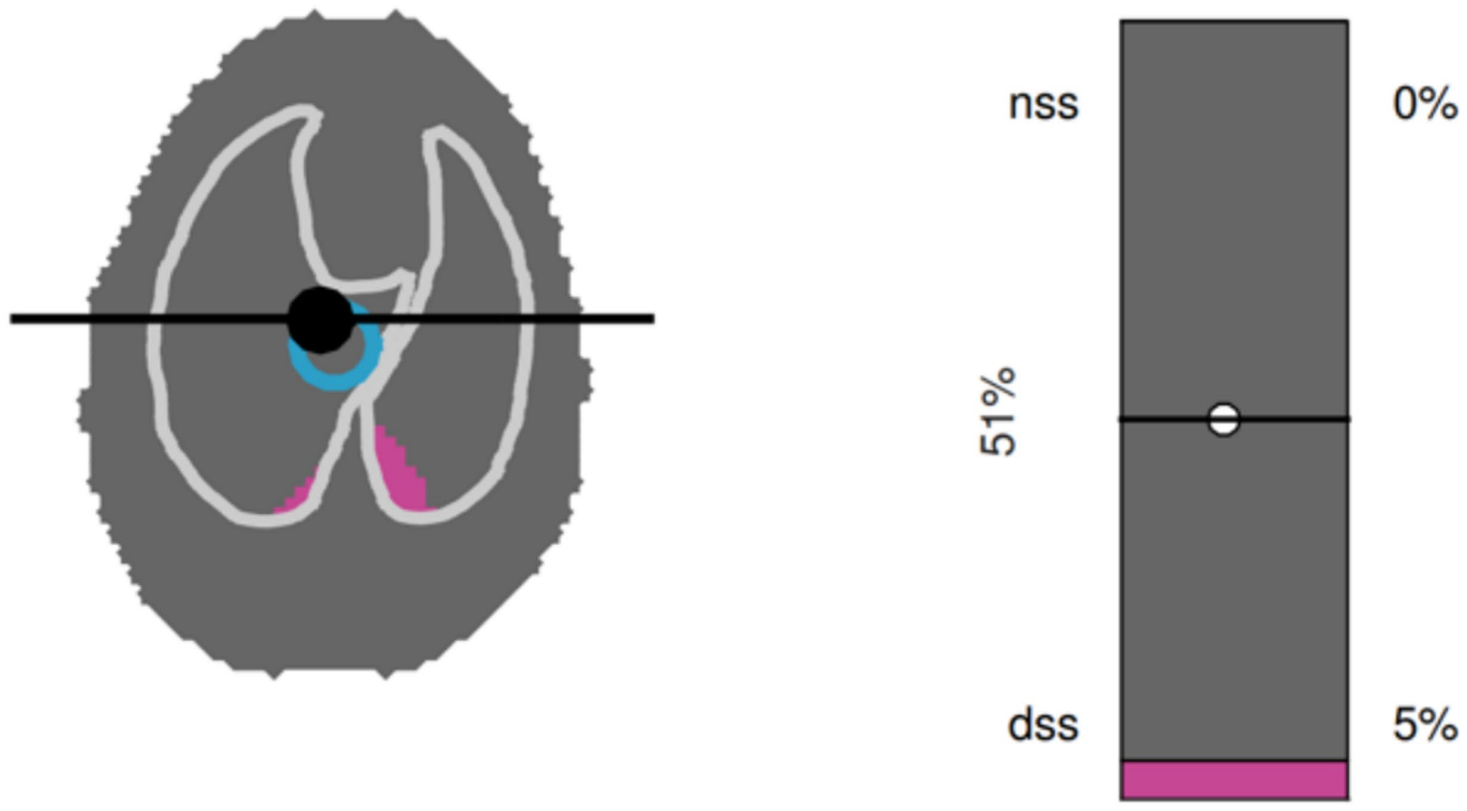
Graphical output from the software used to analyze electrical impedance tomography data in a dorsally recumbent dog. A single breath is shown. Left panel: Silent spaces are shown in purple. In the example shown, only dependent silent spaces (DSS), but not non-dependent silent spaces (NSS) are present. The blue circle represents the center of ventilation (CoV). Right panel: Numerical representation of NSS, DSS and CoV.

The following measurements were taken at T0 and T1:

- Inspiratory apnea CT images (Brilliance CXT 16, Philips, Zurich, Switzerland) of the whole thorax with a slice thickness of 1 mm were acquired. The inspiratory hold pressure was 5 cmH_2_O. Scan parameters were 120 kVp, 240 mA/Rotation, tube rotation 0.75 s, field of view adjusted to chest dimensions, collimator pitch 0.688 and matrix 768×768 (Figure 2).
- Volumetric capnography was recorded using the NICO_2_® capnograph (Respironics, Wallingford, CT). This combined airway flow and CO_2_ mainstream sensor was placed between the Y-piece of the breathing circuit and the endotracheal tube. Data were recorded for 1 min using the dedicated software Datacoll (Respironics, Wallingford, United States). The NICO_2_® was calibrated following manufacturers guidelines at the beginning of each anesthesia event.
- Single-plane EIT measurements were performed with a modified EIT Pioneer-Set (Sentec, formerly Swisstom, Landquart, Switzerland), using a skip-4 driving pattern. A custom-made 32-electrode EIT belt was placed around the thorax over a clipped transversal area; the anatomical position was identified as follows: the length of the sternum was measured and multiplied by 0.17. The resulting distance was measured from the caudal aspect of the xyphoid cranially and the belt positioned at this location (25). Electrically low-conductive ultrasound gel was poured between skin and belt to improve skin-electrode contact. EIT data were acquired at a rate of 46 scans per second. To superimpose contourless EIT images on anatomical landmarks, finite element models (FEM) that had been built for each individual dog in a separate research study were used. Briefly, for the construction of the FEM, CT slices within the EIT electrode plane of each dog were isolated and heart, lung and thorax contours delineated using an open-source software (ITK-SNAP) (26). An algorithm [Graz consensus reconstruction algorithm for EIT (GREIT)] was used to reconstruct EIT images. Further details on EIT technology and image reconstruction can be found elsewhere (10). A representative image of CoV, DSS and NSS output by the software is given in Figure 3.
- Additionally, a blood sample was drawn anaerobically from the arterial catheter into a pre-heparinized syringe and analyzed immediately (Rapidpoint, Siemens, Zurich) for evaluation of PaO_2_ and PaCO_2_.

### 2.5 Data analysis

CT images were reconstructed in a lung window algorithm. The DICOM files were then exported to a CT analysis software (Maluna version 3.14, University of Göttingen) on a separate workstation and reviewed (window width: 1600 Hounsfield Units [HU], window level: - 600 HU). Lung fields and airways from the most cranial to the most caudal extents of the lung parenchyma were included in the analysis. First, the regions of interest (i.e., the lung parenchyma and airways) at each CT slice were manually delineated and then analyzed for percentages of lung volume of voxels that were hyper- normally, poorly or not aerated. We arbitrarily defined hyper-aerated (V_hyper_,) as −1,000 to −901 HU, normally aerated (V_normal_) as −900 to −501 HU, poorly aerated (V_poor_) as −500 to −1 HU and non-aerated (V_non_) as 0 to 200 HU. The degree of aeration was color-coded (Figure 3).

Raw CO_2_ and flow data recorded with the NICO_2_® were used to construct VCap as previously described using a custom-built software based on a functional approximation by a Levenberg–Marquardt algorithm (41). Bohr’s dead space as a ratio to tidal volume (VD_Bohr_/VT), airway dead space as a ratio to tidal volume (VD_aw_/VT), alveolar tidal volume (VT_alv_), alveolar dead space (VD_alv_), the ratio of alveolar dead space over alveolar tidal volume (VD_alv_/VT_alv_) and the elimination of CO_2_ per breath (VTCO_2,br_) were calculated from breaths taken over 1 min.

For calculation of EIT variables, 10 consecutive breaths were analyzed, from which CoV, DSS and NSS were calculated. The CoV was determined as a percentage of ventro-dorsal extension of the lung region (10). Percentages of DSS and NSS were calculated based on pixels with ventilation-induced impedance changes below 10% of the maximum pixel value found (10).

### 2.6 Statistics

The number of enrolled animals was based on a power analysis performed after a convenience sample of 6 dogs based on the parallel study in which the dogs were enrolled (23). When V_hyper_ and V_normal_ were considered the variable of interest, a number of 6 animals per group was required. We chose to enroll 9 dogs in order to compensate for potential missing data. Demographic data, the distribution of ventilation on CT, and data gathered from VCap, EIT, and blood gas analysis were tested for normality using the Shapiro–Wilk statistic and transformation was performed when required and mean ± standard deviation or median were calculated.

The first aim was to evaluate the effect of PEEP and RM on healthy lungs as demonstrated by anatomical and functional monitoring. The second aim was to determine whether a relationship existed between the CT measurements and the functional monitoring parameters, accounting for any modulation of that relationship by the ventilation method or measurement time, using ANCOVA linear regression. A mixed effect model was used, which first explored the categorical covariates of method and measurement and their interaction with CT to determine the equality of the slopes of the covariates. If the slopes were the same (no significant interaction), the interaction term for the covariates was excluded from the model; if the slopes were unequal it was included. Re-parameterization of the model to obtain the estimates of the slopes for the covariates directly was performed. Significance of estimates and models was considered at *p* < 0.05. PROC UNIVARIATE and PROC MIXED (SAS 9.4, SAS Institute, Cary, NC) were used.

## 3 Results

All dogs finished all three study periods. Technical malfunctions required the exclusion of CT data of two dogs from group PEEP and one dog from group ZEEP at both time points, and the exclusion of EIT data from 1 dog in group PEEP at T0. Descriptive statistics for CT, EIT, and VCap data as well as results of arterial blood gas analysis for all 3 groups (ZEEP, PEEP, RM) at both time points (T0, T1) are given in Table 1.

**Table 1:** Results of computed tomography (CT), electrical impedance tomography (EIT) and volumetric capnography (VCap) data, and blood gas analysis in nine Beagle dogs before (T0) and after (T1) either continued ventilation with zero end-expiratory pressure (ZEEP), switching to 5 cmH_2_O of positive end-expiratory pressure (PEEP) or performing a recruitment maneuver, followed by PEEP (RM).

Variable	ZEEP	PEEP	RM	ZEEP	PEEP	RM
	T0			T1		
V_hyper_ (%)	4.98 ± 1.38 *n* = 8	4.84 ± 1.26 *n* = 7	5.87 ± 2.05^a^ *n* = 9	4.97 ± 1.30^*^ n = 8	6.21 ± 2.01^Ѳ^ *n* = 7	11.33 ± 4.32^a,*,Ѳ^ *n* = 9
V_normal_ (%)	89.38 ± 1.22 *n* = 8	88.72 ± 1.72 *n* = 7	87.89 ± 2.29^b^ *n* = 9	89.07 ± 1.52 *n* = 8	88.34 ± 2.06^Ԃ^ *n* = 7	84.96 ± 3.86^b,Ԃ^ *n* = 9
V_poor_ (%)	4.81 ± 1.32 *n* = 8	5.61 ± 1.49 *n* = 7	5.39 ± 2.14^c^ *n* = 9	5.12 ± 1.57^§^ *n* = 8	4.62 ± 1.57^Թ^ *n* = 7	3.05 ± 0.57^c,§,Թ^ *n* = 9
V_non_ (%)	0.83 ± 0.08 *n* = 8	0.83 ± 0.10 *n* = 7	0.85 ± 0.11^d^ *n* = 9	0.84 ± 0.09*^ψ^* *n* = 8	0.83 ± 0.18^Ճ^ *n* = 7	0.66 ± 0.09^d,ψ,Ճ^ *n* = 9
VD_Bohr_/VT	0.71 ± 0.02	0.71 ± 0.02^e^	0.71 ± 0.03	0.70 ± 0.02 ^*ω*, ۩^	0.72 ± 0.02^e,۩^	0.71 ± 0.03^ω^
VD_aw_/VT	0.67 ± 0.03	0.68 ± 0.05^f^	0.67 ± 0.05^g^	0.67 ± 0.03^*ϰ*, ᴥ^	0.71 ± 0.05^f,ᴥ^	0.71 ± 0.06^g,ϰ^
VT_alv_ (ml)	41.64 ± 7.57	41.78 ± 9.64^n^	41.98 ± 10.08^m^	42.75 ± 7.50^»,˄^	37.78 ± 9.40^n,˄^	37.56 ± 11.50^m,»^
VD_alv_ (ml)	4.66 ± 2.44	4.73 ± 3.43	4.64 ± 3.75	5.29 ± 2.48͆	4.26 ± 1.79	3.08 ± 3.41͆
VD_alv_/VT_alv_	0.10 ± 0.06	0.11 ± 0.06	0.10 ± 0.07^h^	0.12 ± 0.05*^ϼ^*	0.08 ± 0.05	0.05 ± 0.06^h,ϼ^
VTCO_2,br_ (ml)	1.72 ± 0.19	1.64 ± 0.30	1.74 ± 0.24^i^	1.77 ± 0.14^ж,‡^	1.56 ± 0.30^‡^	1.48 ± 0.25^i,ж^
NSS (%)	0.02 ± 0.06 *n* = 9	0.20 ± 0.44 *n* = 8	0.81 ± 2.44 *n* = 9	0.30 ± 0.56 *n* = 9	0.03 ± 0.08 *n* = 9	0.23 ± 0.40 *n* = 9
DSS (%)	5.83 ± 3.36 *n* = 9	6.88 ± 2.91 *n* = 8	6.52 ± 2.57 *n* = 9	6.92 ± 3.40 *n* = 9	5.12 ± 2.40 *n* = 9	5.46 ± 2.93 *n* = 9
CoV (%)	54.82 ± 2.29 *n* = 9	55.28 ± 2.35^j^ *n* = 8	54.35 ± 3.58^k^ *n* = 9	54.53 ± 2.39^ђ,⁋^ *n* = 9	56.70 ± 1.81^j,⁋^ *n* = 9	56.21 ± 3.13^k,ђ^ *n* = 9
PaO_2_ (kPa)	76.02 ± 2.92	77.21 ± 2.56	76.7 ± 3.77	74.76 ± 2.65^Ѯ^	75.00 ± 2.69	76.35 ± 3.86^Ѯ^
PaO_2_ (mmHg)	570.27 ± 21.93	579.23 ± 19.22	575.42 ± 28.26	560.83 ± 19.88^Ѯ^	562.61 ± 20.17	572.74 ± 28.93^Ѯ^
PaCO_2_ (kPa)	5.62 ± 0.95	5.45 ± 0.52	5.69 ± 0.86^l^	5.57 ± 0.81	5.68 ± 0.57^֍^	5.31 ± 0.83^l,֍^
PaCO_2_ (mmHg)	42.19 ± 7.12	40.92 ± 3.88	42.69 ± 6.48^l^	41.76 ± 6.10	42.59 ± 4.27^֍^	39.84 ± 6.23^l,֍^

CT variables: Hyperaerated (V_hyper_), normally aerated (V_normal_), poorly aerated (V_poor_) and non-aerated (V_non_) volume. VCap variables: Bohr’s physiologic dead space (VD_Bohr_/VT), airway dead space to tidal volume ratio (VD_aw_/VT), alveolar dead space to alveolar tidal volume ratio (VD_alv_/VT_alv_), and elimination of CO_2_ per breath (VTCO_2,br_). EIT variables: non-dependent silent spaces (NSS), dependent silent spaces (DSS), and center of ventilation (CoV). Blood gas variables: Arterial partial pressure of oxygen (PaO_2_), and arterial partial pressure of carbon dioxide (PaCO_2_) Data are presented as mean ± SD. *n* denotes the number of dogs for which data were available (if *n* not provided, data were available for all dogs). The same letters and symbols in superscript indicate a statistically significant difference.

### 3.1 Comparing ventilation modes

**Figure 4:**
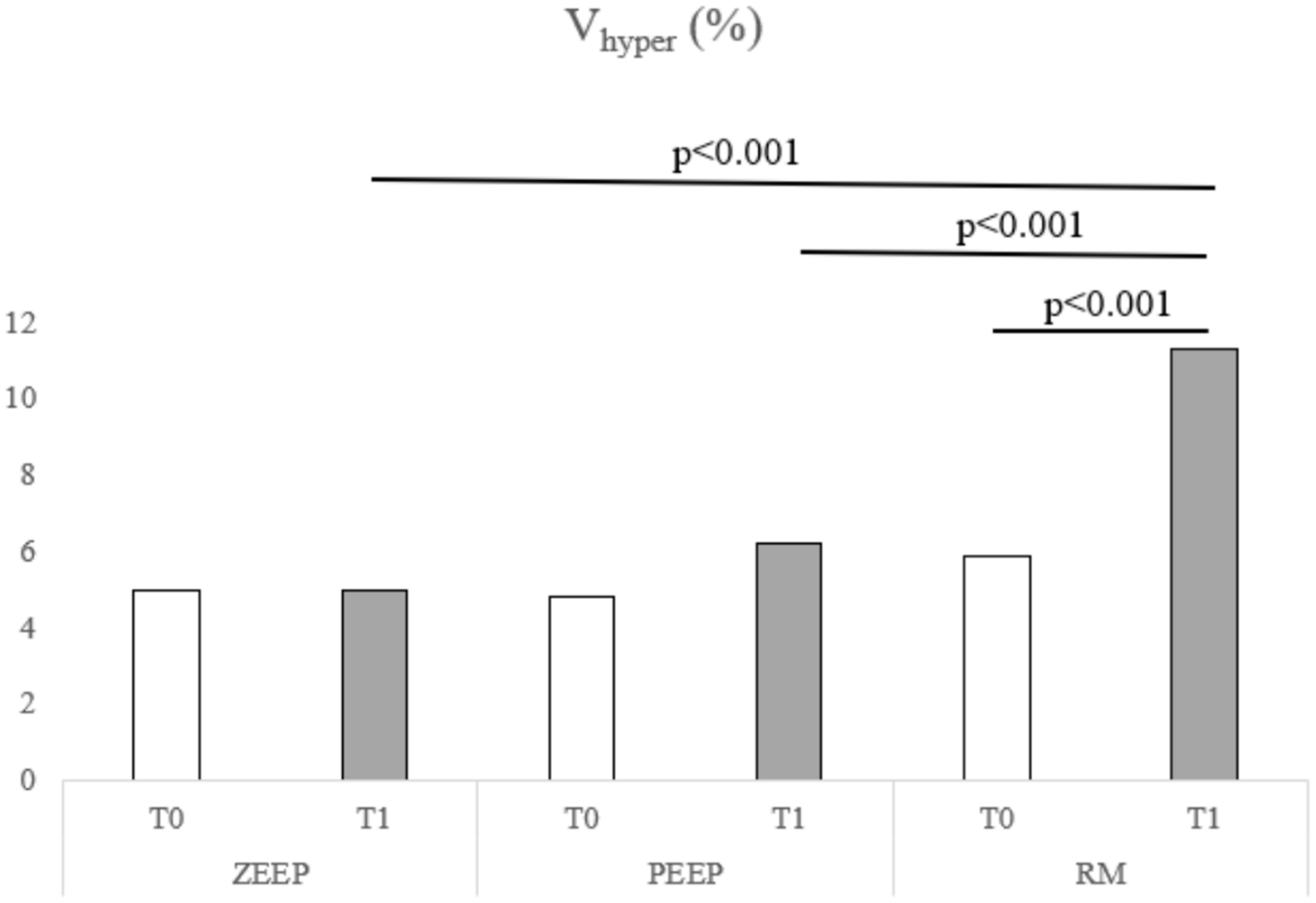
Distribution of V_hyper_ over time in all groups (ZEEP, zero-end-expiratory pressure; PEEP, positive end-expiratory pressure; RM, recruitment maneuver).

**Figure 5:**
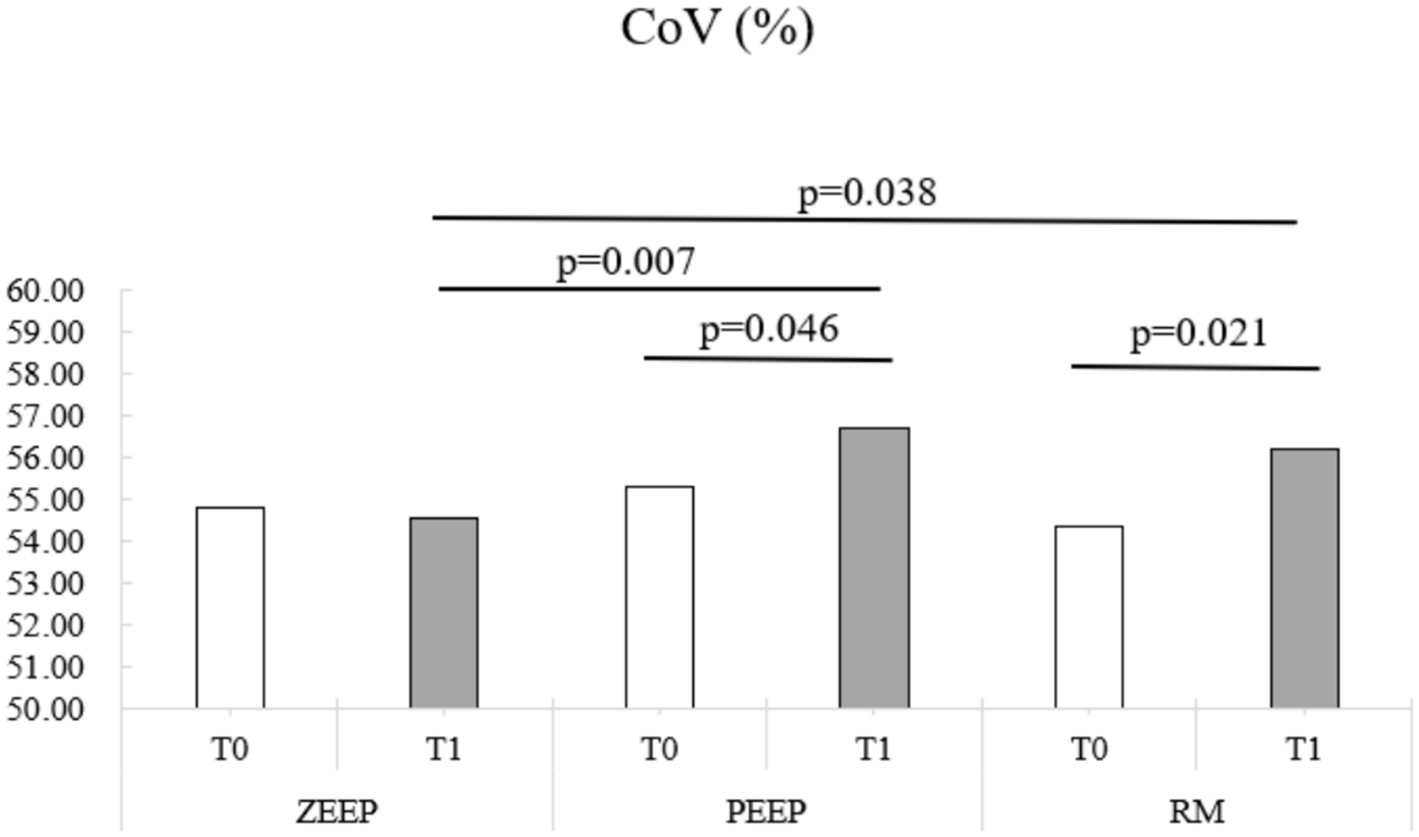
Distribution of the center of ventilation (CoV) over time in all groups (ZEEP, zero-end-expiratory pressure; PEEP, positive end-expiratory pressure; RM, recruitment maneuver).

When evaluating the different ventilation modes between time points and between each other, the following results were obtained:

- Computed tomography revealed an increase in the percentage of V_hyper_ in the RM group at T1 compared to T0 (*p* < 0.001). V_hyper_ was also higher in the RM group at T1 compared to ZEEP (*p* < 0.001) and PEEP at the same time point (*p* < 0.001) (Figure 4). The percentage of V_normal_ was lower in RM at T1 compared to T0 (*p* = 0.001), and lower in the RM group compared to the PEEP group at T1 (*p* < 0.001). The percentage of V_poor_ was lower in RM at T1 compared to T0 (*p* < 0.001). The percentage of V_poor_ was also lower in the RM group than in the ZEEP and PEEP groups at T1 (*p* < 0.001 and *p* = 0.002, respectively). The percentage of V_non_ was lower in RM at T1 compared to RM at T0 (*p* < 0.001), and it was also lower in the RM group than in the ZEEP and PEEP groups at T1 (*p* < 0.001 and *p* = 0.001, respectively).
- Volumetric capnography showed that VD_Bohr_/VT was higher in PEEP at T1 compared to PEEP at T0 (*p* = 0.029), which was not the case for the RM group (*p* = 0.289). However, VD_Bohr_/VT was higher in the RM and PEEP groups compared to the ZEEP group at T1 (*p* = 0.039 and *p* < 0.001, respectively). VD_aw_/VT was higher in in the RM group at T1 compared to T0 (*p* = 0.005), and in the PEEP group at T1 compared to T0 (*p* = 0.003). Furthermore, it was higher in RM and PEEP compared to ZEEP at T1 (*p* = 0.002 and p = 0.002, respectively), but there was no difference between PEEP and RM at T1. VT_alv_ was significantly lower in RM compared to ZEEP at T1 (*p* = 0.006), and in PEEP compared to ZEEP at T1 (*p* = 0.008). It was also significantly lower in RM at T1 compared to T0 (*p* = 0.019) and in PEEP at T1 compared to T0 (*p* = 0.034). VD_alv_ was significantly smaller in RM compared to ZEEP at T1 (*p* = 0.041). VD_alv_/VT_alv_ decreased in the RM group at T1 compared to T0 (*p* = 0.018), and it was lower in RM compared to ZEEP at T1 (*p* = 0.001). VTCO_2,br_ was lower in the RM group at T1 compared to T0 (*p* < 0.001), and it was also lower in the RM group and PEEP groups compared to the ZEEP group at T1 (*p* < 0.001 and *p* < 0.001).
- EIT values for NSS or DSS did not change significantly over time or with ventilation mode. CoV increased in the RM group at T1 compared to T0 (*p* = 0.021) and in the PEEP group at T1 compared to T0 (*p* = 0.046). It was also higher in the RM group and the PEEP group compared to the ZEEP group at T1 (*p* = 0.038 and *p* = 0.007, respectively), indicating a shift of ventilation towards the dorsal (dependent) parts of the lungs in these two groups, while no change was observed in the control group (Figure 5).
- PaO_2_ did not change between T1 and T0 in any group. Compared to the control group however, PaO_2_ was higher in the RM group at T1 (*p* = 0.033). PaCO_2_ decreased at T1 compared to T0 in the RM group (*p* = 0.012) and was also lower in the RM group compared to the PEEP group at T1 (*p* = 0.016).

### 3.2 Comparing monitoring systems

Comparing the various monitoring tools with each other, a positive interaction was found for V_hyper_ with VD_aw_/VT (*p* = 0.004) and VTCO_2,br_ (*p* = 0.002), while VD_alv_/VT_alv_ (*p* = 0.004) and NSS (*p* = 0.050) decreased when V_hyper_ increased. V_normal_ had a positive association with VTCO_2,br_ (*p* = 0.002) and a negative association with PaO_2_ (*p* = 0.030). No other significant interaction was found between the anatomical CT values and the functional VCap, EIT and blood gas results.

## 4 Discussion

This study provides valuable insights into the effects of different ventilation modes - ZEEP, PEEP, and RM - on lung function in healthy dogs, using a range of monitoring tools to compare functional and anatomical assessments. The findings underscore the differential impact of recruitment maneuvers (RM) and positive end-expiratory pressure (PEEP) on lung ventilation patterns, highlighting specific shifts in ventilation distribution, dead space, and gas exchange.

### 4.1 Effects of ventilation modes on lung function

Overall, the pre-intervention lung status was better than anticipated, with less than 1% of non-aerated lung tissue and approximately 5% of poorly aerated lung regions, which is comparable to what has been demonstrated in healthy, spontaneously breathing humans (27) and distinctly less than what has been reported in dogs during mechanical ventilation (28). This suggests that a recruitment maneuver (RM) might not have been clinically necessary. However, the aim of the study was to investigate the physiological effects of RM on the lungs, even though it was not clinically indicated, as this approach allowed us to better understand its impact under controlled conditions in healthy lungs.

The recruitment maneuver nearly doubled the hyperventilated lung volume to over 10% at T1, compared to baseline (T0). Concurrently, RM decreased the volumes of poorly ventilated and non-ventilated lung regions, as visualized on CT. Although the extent of recruitment was lower than the increase in V_hyper_ this indicates that RM can reduce absorption atelectasis or under-ventilation in healthy lung tissue during anesthesia. Notably, the primary finding was a significant reduction in normally aerated lung volume after RM, suggesting that hyperinflation surpassed recruitment, thus cautioning against the use of RM in healthy canine lungs. This finding is particularly relevant when considering the potential for an inflammatory response associated with lung tissue overstretch, as observed in studies with horses (5). In contrast, another study in healthy dogs also found a decrease in non-aerated areas with RM (performed as sustained inflation), but observed an increase in V_normal_ rather than a decrease, with no changes in V_hyper_ (29) which indicates that also the protocol for conducting a recruitment maneuver has an influence on the outcome. Several methods of performing recruitment maneuvers in people and animals are described in the medical literature (13, 29–34). We chose a stepwise recruitment technique used in humans (24), as in people it is no longer recommended to perform sustained inflation maneuvers (30), and the described technique is customary at one of the authors’ institutions. There is currently no consensus on which stepwise recruitment approach is superior to another (30).While PEEP also reduced V_poor_ over time, this reduction did not achieve statistical significance. The increase in V_hyper_ during PEEP was also more modest, remaining below 2%. PEEP therefore might be the safer strategy to counteract atelectasis in healthy dogs during anesthesia.

Volumetric capnography showed that the increase in V_hyper_ was largely attributable to elevated airway dead space ventilation in both the RM and PEEP groups at T1 compared to T0, without significant differences between these groups. Although both RM and PEEP increased dead space, RM may facilitate more effective alveolar ventilation, as shown by a 5% reduction in alveolar dead space fraction in the RM group at T1, especially when compared to ZEEP, which showed an increase in this fraction over the same interval. However, VTCO_2,br_ was significantly lower in both RM and PEEP at T1. Given that VCO_2,br_ can serve as an indirect measure of cardiac output in healthy lungs (9), this decrease suggests an expected adverse effect of high mean airway pressure on cardiovascular function.

In comparing our data to other veterinary studies, overall, our VCap parameters were considerably different from what is reported in other studies on healthy dogs, with dead space being higher and VTCO_2,br_ being lower (35–38). The reason for this is unclear.

### 4.2 EIT and regional ventilation distribution

Electrical impedance tomography showed increased ventilation towards the dorsal, dependent regions (CoV) in both RM and PEEP groups at T1. This suggests that both RM and PEEP promoted a more favorable distribution of ventilation, potentially counteracting gravitational effects on lung perfusion and improving gas exchange. The lack of significant changes in NSS and DSS across time points and ventilation modes, however, is due to the high standard deviation seen in our dogs and indicates that EIT’s capacity to detect regional shifts may be more pronounced in specific parameters like CoV. This shift in CoV is in contrasts with other studies where RM of 20 cmH_2_O did not cause significant changes in CoV in dogs (12, 13).

### 4.3 Arterial blood gas analysis and gas exchange

Interestingly, PaO_2_ levels were generally higher in the RM group compared to the control group at T1. Notably, when comparing PaO_2_ at T1 to baseline across the groups, PaO_2_ showed a decrease in all groups without reaching statistical significance, with the smallest decrease observed in the RM group. This decrease in PaO_2_ likely reflects the effects of anesthesia over time, which seem to be blunted with RM. However, with PaO_2_ levels remaining above 73 kPa (550 mmHg), a 1.33 kPa (10 mmHg) drop is clinically negligible and possibly within measurement error. It would have been valuable to assess the impact of PEEP and RM on oxygenation at an FiO_2_ of 0.21, but FiO_2_ was set at 1.0 intentionally to induce absorption atelectasis.

A similar observation was made for PaCO_2_, which showed a statistically significant but clinically minor reduction following RM, likely due to increased minute ventilation during the maneuver that still persisted 10 min post-RM.

### 4.4 Comparative efficacy of monitoring techniques

When comparing the different monitoring modalities, we observed specific correlations between CT, VCap, and blood gas analysis, but few interactions were found with EIT values.

The increase in V_hyper_ observed on CT correlated positively with the VD_aw/_VT ratio, suggesting that the overdistension effects of PEEP and RM influenced airway geometry and gas flow. This likely resulted from a distal shift of the interface between airway dead space and alveolar ventilation, with smaller airway diameters increasing rather than alveolar distension. This interpretation aligns with Fletcher’s findings, which described how airway adjustments in response to pressure can alter ventilation distribution without necessarily expanding alveolar spaces (39).

While the center of ventilation was a valuable EIT variable for evaluating a change in dorso-ventral ventilation distribution, other EIT variables showed limited correlation with CT and VCap data, likely due to EIT’s lower spatial resolution in detecting the small non- and poorly ventilated lung areas in our healthy dogs after a relatively short period of anesthesia. Although an increase in V_hyper_ to over 10% was expected to be detected by the NSS metric, especially as we used individualized finite element models for EIT analysis, this was not observed. Notably, comparing EIT and VCap as functional monitoring tools shows NSS and VD_alv_/VT_alv_ align directionally after RM, whereas the anatomical CT measure V_hyper_ contrasts with this. This underscores the need for caution when comparing anatomical imaging modalities with functional lung monitoring techniques.

While the positive correlation between normally aerated lung tissue and VTCO_2,br_ makes sense as it shows that the lungs become more efficient in eliminating CO_2_ (9), the positive correlation of V_hyper_ and VTCO_2,br_ is unexplained. Additionally, the inverse correlation between V_normal_ and PaO_2_, which is contradictory, indicates that blood gasses assess gas exchange dynamics beyond anatomical status alone, again underscoring the need for caution when comparing anatomical imaging like CT with functional measures of lung performance.

### 4.5 Limitations

This study faced several key limitations that may have impacted our findings. First, we were able to induce only minimal atelectasis and areas of poor aeration prior to initiating the ventilation strategies. As such, there was limited potential for improvement, which may have minimized observable effects. To induce atelectasis, an FiO_2_ of 1.0 was used to promote absorption atelectasis, as this has been shown to occur in other studies within a few minutes (40). However, this high FiO_2_ resulted in less than 1% non-aerated and around 5% poorly aerated lung areas, potentially insufficient to reveal notable changes in the functional monitoring modalities with the ventilation strategies applied. If a lower FiO_2_ had been chosen, it is possible that more significant shifts in PaO_2_ and greater efficacy of the ventilation strategies might have been detectable.

Additionally, the duration of each ventilation intervention was brief, limiting our ability to observe potential longer-term effects on lung aeration and function. These factors underscore the challenges in detecting subtle physiological responses in healthy lung models and suggest that future studies with prolonged ventilation times and alternative FiO_2_ levels might yield further insights into the effects of ventilation strategies on lung function in minimally compromised lungs.

## 5 Conclusion

Based on our data, applying a recruitment maneuver in healthy lungs during short time mechanical ventilation seems to carry more risks (reduction of normally aerated lungs, increase in hyperaerated lungs, reduction in VTCO_2,br_), than benefits (reduction of poorly or non-ventilated areas, shifting of the focus of ventilation towards dependent lung areas), even though it might be more effective than PEEP alone in blunting decrease in PaO_2_ over time, at least when 100% oxygen is applied. PEEP may be gentler on healthy lungs, as hyperaerated lung areas do not increase, but also less effective, as it only shifts CoV dorsally without reducing poorly or non-aerated lung areas. Although the decrease for VTCO_2,br_ is concerning for either a decrease in healthy lung tissue or pulmonary perfusion also during PEEP, we conclude that PEEP is likely the preferable ventilation technique over RM in healthy lungs mechanically ventilated at an FiO_2_ of 1.0.

Based on our data, functional lung monitoring should be cautiously used to directly compared to anatomical monitoring by CT for the interpretation of changes after ventilation maneuvers in healthy lungs.

## Data Availability

The raw data supporting the conclusions of this article will be made available by the authors, without undue reservation.
